# Supplementary material for: RNA-Seq analysis and annotation of a draft blueberry genome assembly identifies candidate genes involved in fruit ripening, biosynthesis of bioactive compounds, and stage-specific alternative splicing
Source: Gigascience. 2015 Feb 13;4:5. doi: 10.1186/s13742-015-0046-9 (PMC4379747; doi:10.1186/s13742-015-0046-9)
Supplement: Additional file 1: Figure S1. — Data processing and analysis pipeline. The computational workflow used to generate blueberry gene models and coding sequences is shown. (A) Sources of gene annotations. (B) Step-wise selection protocol used to create the nonredundant gene set. Figure S2. Expression pattern of candidate ethylene biosynthesis genes. Expression of (A) SAM synthase, (B) ACC synthase and (C) ACC oxidase genes are shown in RPKM. (D) Enzymes and intermediates in ethylene biosynthesis pathway. Figure S3. Expression pattern for candidate bixin and dhurrin biosynthetic genes. (A) Bixin biosynthesis as annotated in PlantCyc as pathway id PWY-5305. The first step is catalyzed by a lycopene cleavage oxygenase, which was not found in the blueberry annotations. Subsequent reactions are catalyzed by enzymes BADH (bixin aldehyde dehydrogenase) and nBMT (norbixin methyltransferase), which were found in blueberry. (B) and (C) Expression of blueberry genes encoding BADH and nBMT are shown on two plots with different y-axis scales as one gene (CUFF.4231) is highly expressed in fruit. (D) Synthesis and breakdown of cyanogenic glycoside dhurrin. (E-J) Expression of genes encoding putative dhurrin synthesis and breakdown enzymes. Figure S4. Stage-specific alternative promoter and three prime ends. (A) Read alignments from mature green and ripe fruit showing an alternative promoter in CUFF.24092, which encodes a conserved protein of unknown function. (B) Coverage graphs showing read density at locus CUFF.32196, encoding a conserved protein of unknown function. Graphs represent the number of reads that overlap the base positions indicated on the coordinates axis track. Figure S5. RNA-Seq coverage graphs illustrating expression of genes involved in synthesis of proanthocyanidins, anthocyanins, or both. Each image shows alignments of ESTs alongside blueberry gene models. (A) CUFF.29797, encoding VcANR, anthocyanidin reductase (B) CUFF.20951, encoding VcUFGT (C) VcDFR (D) VcMYBPA1a (E) VcMYBPA1b. [file 13742_2015_46_MOESM1_ESM.pdf]

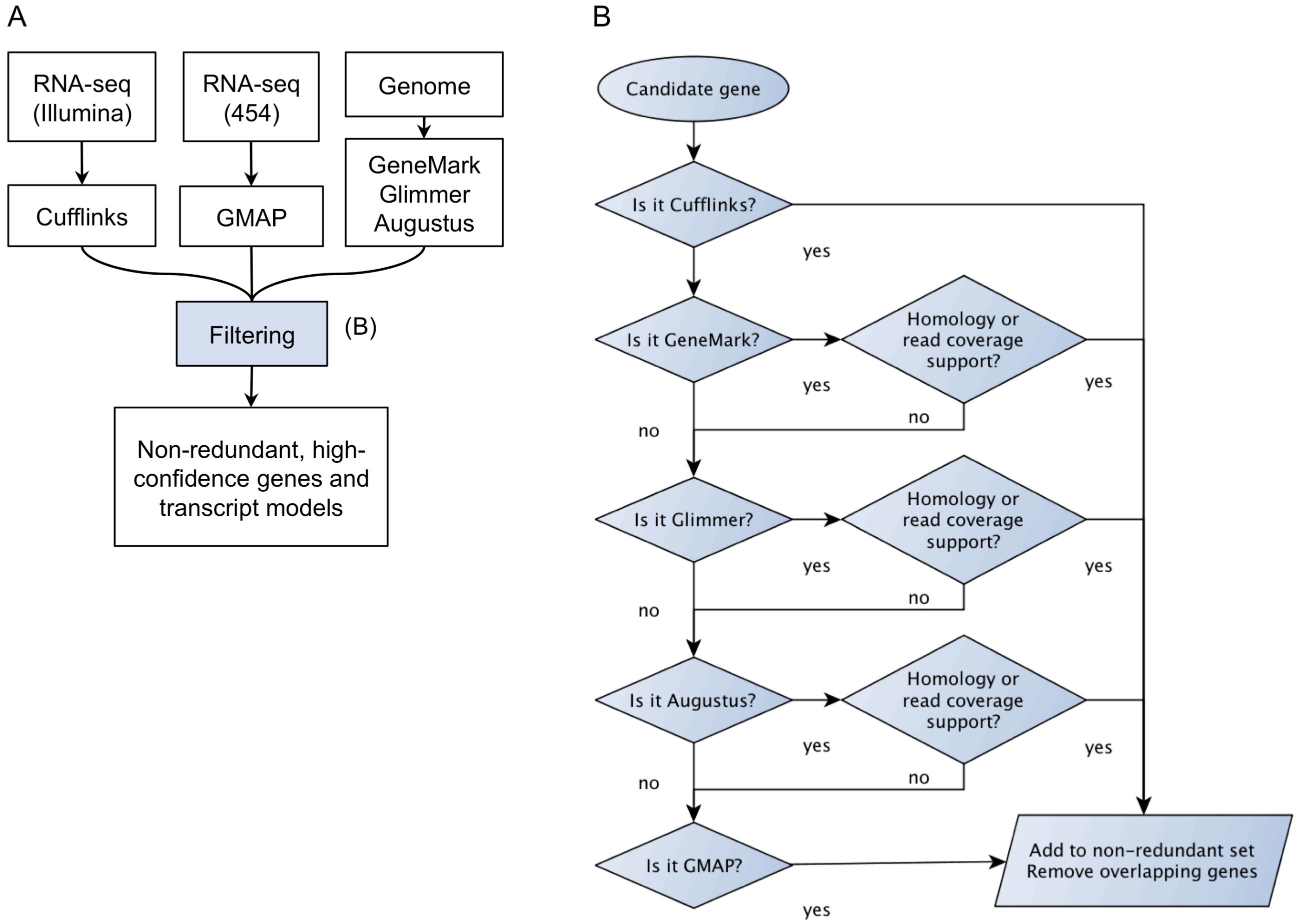

Figure 1. Data processing and analysis pipeline. The computational workflow used to generate blueberry gene models and coding sequences is shown. (**A**) Sources of gene annotations. (**B**) Step-wise selection protocol used to create the non-redundant gene set.

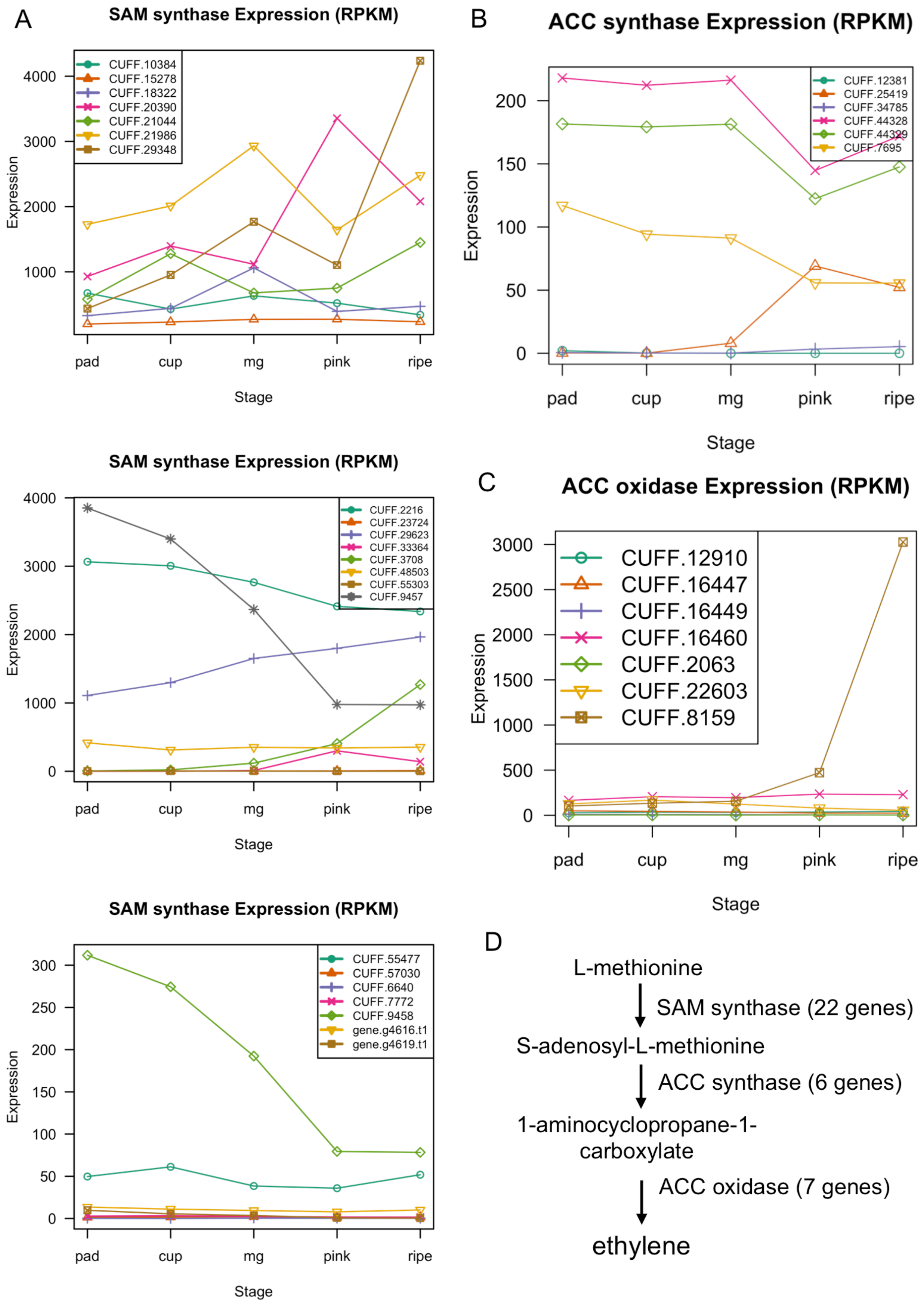

Supplemental Figure 2. Expression pattern of candidate ethylene biosynthesis genes. Expression of **(A)** SAM synthase, **(B)** ACC synthase and **(C)** ACC oxidase genes are shown in RPKM. **(D)** Enzymes and intermediates in ethylene biosynthesis pathway.

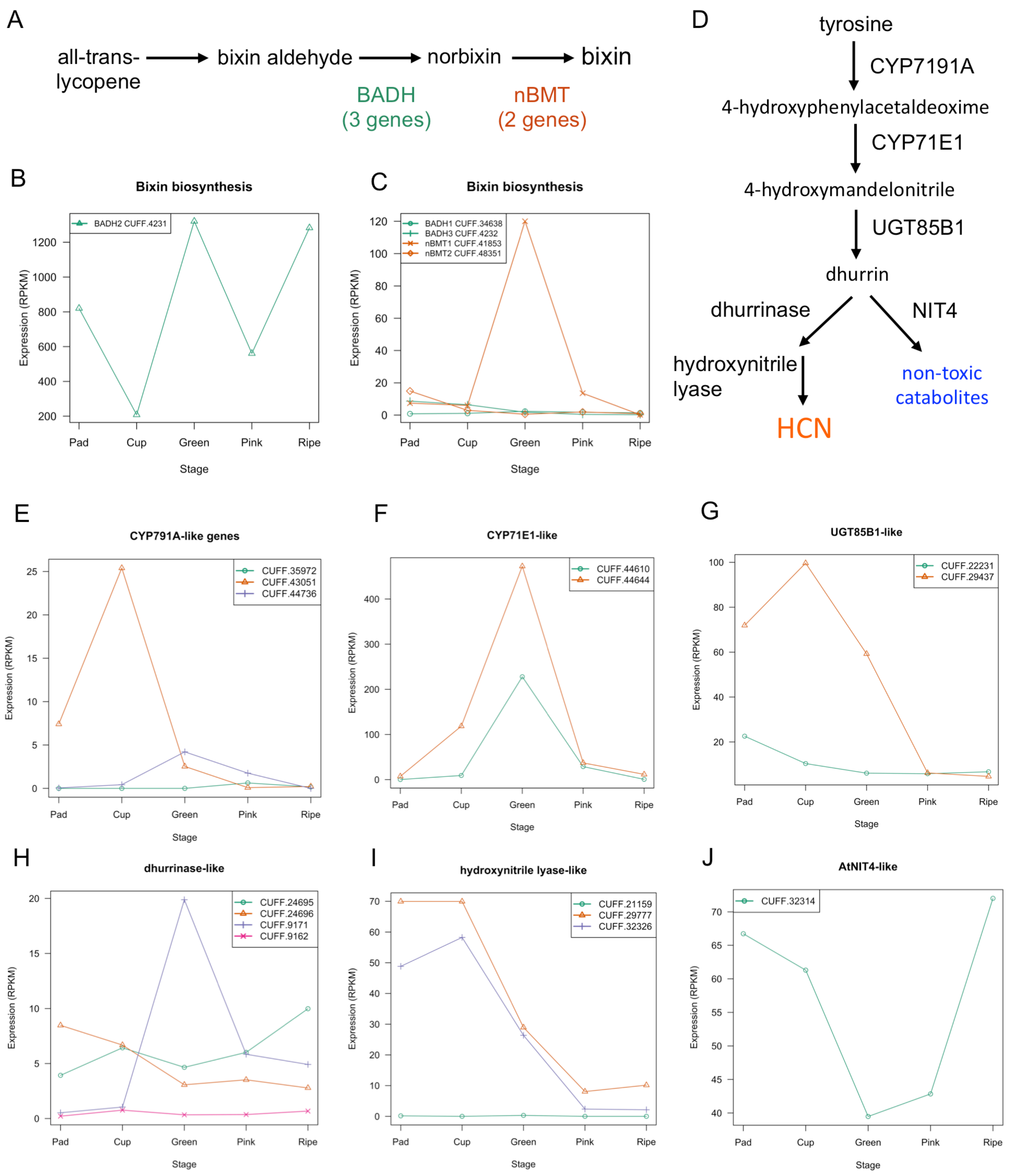

Supplemental Figure 3. Expression pattern for candidate bixin and dhurrin biosynthetic genes. **(A)** Bixin biosynthesis as annotated in PlantCyc as pathway id PWY-5305. The first step is catalyzed by a lycopene cleavage oxygenase, which was not found in the blueberry annotations. Subsequent reactions are catalyzed by enzymes BADH (bixin aldehyde dehydrogenase) and nBMT (norbixin methyltransferase), which were found in blueberry. (**B**) and (**C**) Expression of blueberry genes encoding BADH and nBMT are shown on two plots with different y-axis scales as one gene (CUFF.4231) is extremely highly expressed in fruit. (**D**) Synthesis and breakdown of cyanogenic glycoside dhurrin. (E-J) Expression of blueberry genes encoding putative dhurrin synthesis and breakdown enzymes.

A

B

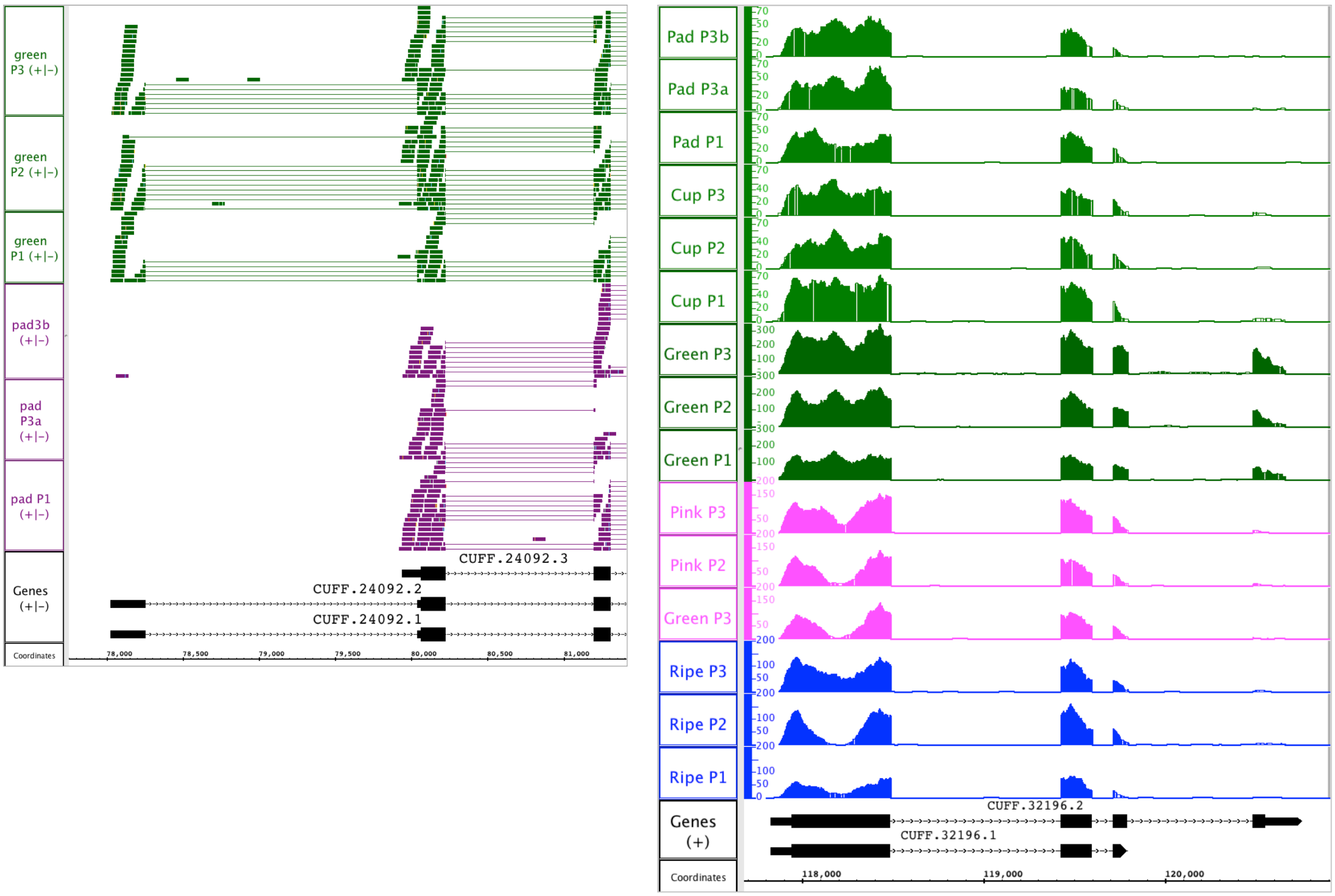

Supplemental Figure 4. Stage-specific alternative promoter and three prime ends. **(A)** Read alignments from mature green and ripe fruit showing an alternative promoter in CUFF.24092, which encodes a conserved protein of unknown function. The direction of transcription is left to right. **(B)** Coverage graphs showing read density at locus CUFF.32196, encoding a conserved protein of unknown function. Direction of transcription is left to right. Graphs represent the number of reads that overlap the base positions indicated on the coordinates axis track.

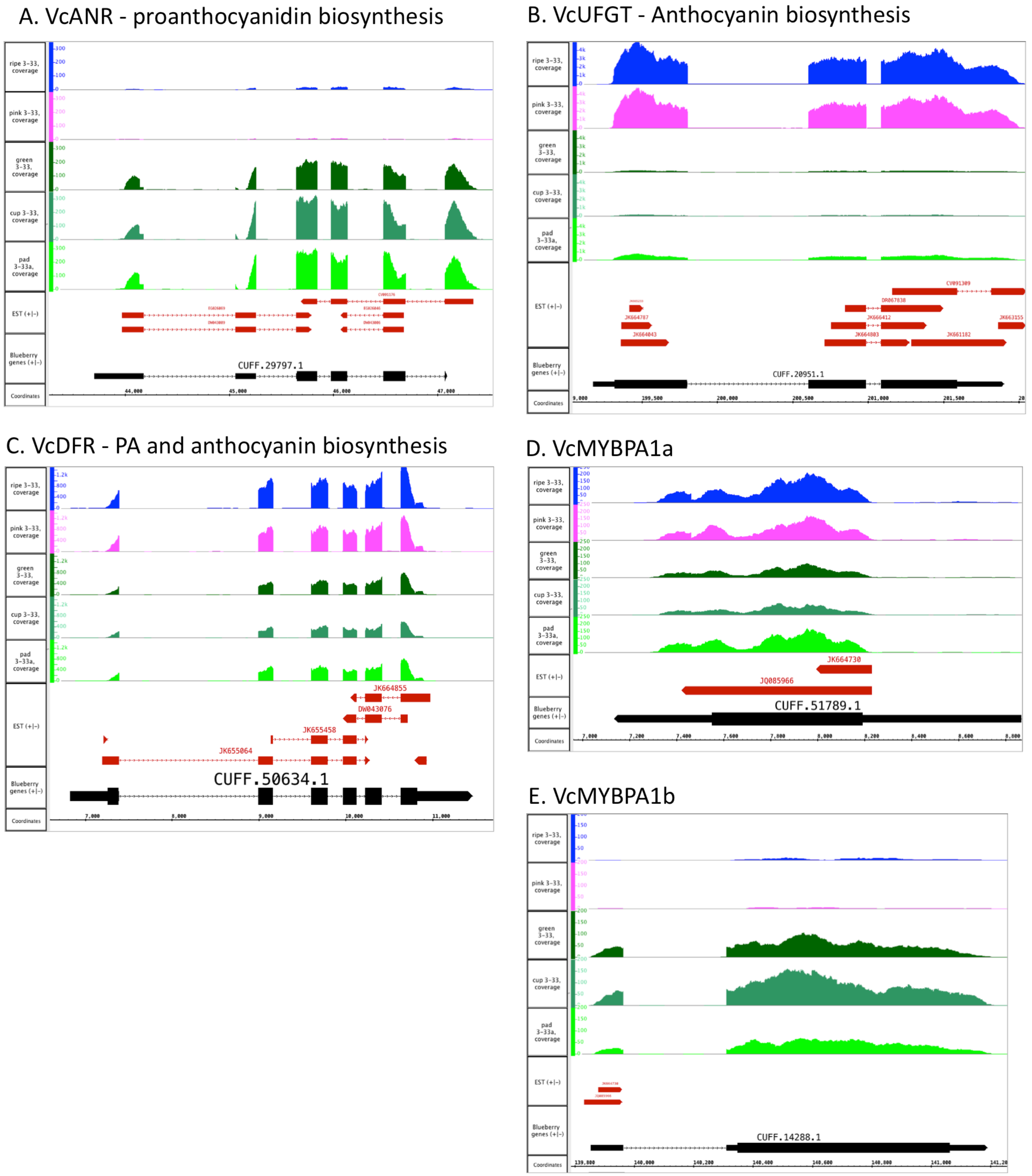

Supplemental Figure 5. RNA-Seq coverage graphs illustrating expression of genes involved in synthesis of proanthocyanidins, anthocyanins, or both. Each image shows alignments of ESTs alongside blueberry gene models. (**A**) CUFF.29797, encoding VcANR, anthocyanidin reductase (**B**) CUFF.20951, encoding VcUFGT, also shown in Figure 7A (**C**) VcDFR (**D**) VcMYBPA1a (**E**) VcMYBPA1b
